# A model of conceptual bootstrapping in human cognition

**DOI:** 10.1038/s41562-023-01719-1

**Published:** 2023-10-16

**Authors:** Bonan Zhao, Christopher G. Lucas, Neil R. Bramley

**Affiliations:** 1 Department of Psychology, University of Edinburgh, Edinburgh, UK; 2 School of Informatics, University of Edinburgh, Edinburgh, UK

**Keywords:** Human behaviour, Computational models, Education, Science, technology and society, Language and linguistics

## Abstract

To tackle a hard problem, it is often wise to reuse and recombine existing knowledge. Such an ability to bootstrap enables us to grow rich mental concepts despite limited cognitive resources. Here we present a computational model of conceptual bootstrapping. This model uses a dynamic conceptual repertoire that can cache and later reuse elements of earlier insights in principled ways, modelling learning as a series of compositional generalizations. This model predicts systematically different learned concepts when the same evidence is processed in different orders, without any extra assumptions about previous beliefs or background knowledge. Across four behavioural experiments (total *n* = 570), we demonstrate strong curriculum-order and conceptual garden-pathing effects that closely resemble our model predictions and differ from those of alternative accounts. Taken together, this work offers a computational account of how past experiences shape future conceptual discoveries and showcases the importance of curriculum design in human inductive concept inferences.

## Abstract (web summary)

Zhao et al. present a model of conceptual bootstrapping through which they model learning complex concepts by recursively combining simpler concepts.

## Main

People have a remarkable ability to develop rich and complex concepts despite limited cognitive capacities. On the one hand, there is abundant evidence that people are bounded reasoners^1–5^, entertain a rather small set of mental options at a time^6–10^ and generally deviate from exhaustive search over large hypothesis spaces^11–15^. On the other hand, these bounded reasoners can develop richly structured conceptual systems^16–18^, produce sophisticated explanations^19–21^ and push forward complex scientific theories^22^. How are people able to create and grasp such complex concepts that seem so far beyond their reach?

Newton gave a famous answer to this question: “If I have seen further, it is by standing on the shoulders of giants”^23^. This reflects the intuition that people are bounded yet blessed with a capacity not just to learn from others, but to extend and repurpose existing knowledge to create new and more powerful ideas. Such ability is taken to be a cornerstone of cognitive development^24^. For instance, by building from atomic concepts of small numbers one, two, three and counting, young children seem to bootstrap to more general and abstract numerical concepts such as successor relationships and the infinite line of real numbers^25^. Via bootstrapping, extant hard-earned knowledge need not be rediscovered every time it is used, saving the learner time and effort in constructing new concepts that build on old concepts. Because of such effective rerepresentation of existing knowledge, people can arrive at rich mental constructs incrementally^26–28^ and grow a hierarchy of concepts naturally through levels of nested reuse^18^.

While bootstrapping is a key idea in theories of learning and development^24^, both behavioural studies that examine bootstrapping directly and cognitive models articulating its mechanisms are relatively rare. Piantadosi et al.^25^ pioneered a line of research that posited bootstrapping in a Bayesian concept-learning framework. However, they focused on the discovery of a recursive function in learning numeric concepts and left open the task of examining bootstrapping as a general model of online inductive inference. Dechter et al. ^29^ formalized the idea that an artificial learner can start with solving simple search problems and then reuse some of the solutions to make progress in more complex problems. This approach later developed into Bayesian library learning, a class of models aimed at extraction of shared functionalities from a collection of programmes^30,31^. These models have successfully solved a variety of tasks and have been shown to capture aspects of human cognition^32,33^. However, these works are primarily aimed at learning optimal libraries or solving challenging test problems rather than explicating how resource limitations interact with the mechanisms of bootstrapping, and how exploiting such interactions may explain human patterns of reasoning errors as well as successes.

Here we provide a computational model of how people bootstrap, and propose an algorithmic mechanism that progressively produces rich concepts, even with limited cognitive resources. Treating the way in which people construct concepts as a computational problem, we model bootstrapping as a process-level learning algorithm^34^ that effectively caches previous learned concepts and reuses them for more complex concepts through principled rerepresentation. To achieve this, we extend standard Bayesian concept-learning frameworks with a dynamic concept library that can be enriched over time, powered by a formalization drawn from adaptor grammars^35,36^. We then design experiments informed by this model to test and measure how people construct complex concepts and how this process adapts to the order in which people encounter, or think about, evidence. We compare this bootstrap learning account with a variety of alternative models of concept learning and demonstrate how a cache-and-reuse mechanism provides an account for human inferential limitations, as well as how it enables us to reach concepts that are initially beyond our grasp, under facilitatory conditions.

## Formalization

**Fig. 1:**
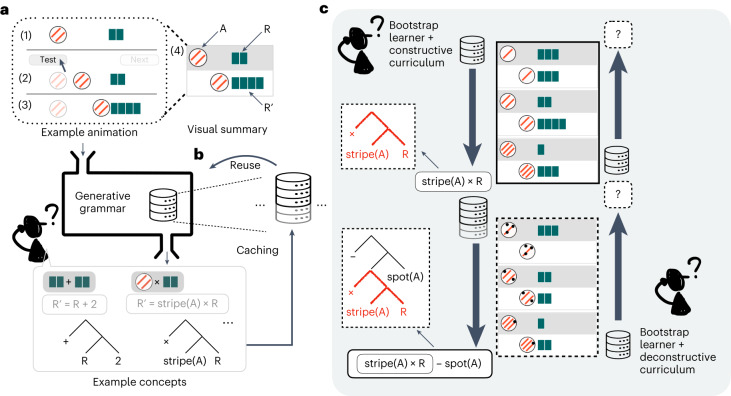
Model and task summaries. **a**, Example causal interaction with (1) causal agent (left, circle) and recipient (right) objects; (2) agent A moves rightward to the recipient R; and (3) on touching, the recipient R changes into its result form R'. The translucent marker is used here only to illustrate the animation. Summary of this animation (4), with grey background showing agent A and recipient R before the causal interaction, and white background representing the agent A and result R' following the causal interaction. **b**, Schematic of the bootstrap learning model. Trees represent example concept programmes. **c**, Example bootstrap learning trajectories over six observations (see main text for explanation).

Consider the causal learning and generalization task depicted in Fig. 1a. An agent object A (called a ‘magic egg’ in our experiments) moves toward a recipient object R (called a ‘stick’) and, on touching each other, agent object A causes changes to the number of segments on recipient object R, producing what we call the result object R'. Here an agent object has two numerical features—a number of stripes and a number of spots—and people are asked to hypothesize about the nature of the causal relationship between agent and recipient objects and the result, or formally, the content of function *f*(stripe(A), spot(A), segment(R)) that produces segment(R'). Without ambiguity, we shorten this to R' ← *f*(stripe(A), spot(A), R).

Despite its apparent simplicity, this task captures a key challenge of concept learning: the space of potential hypotheses is infinite. For instance, it could be that object A adds two segments to recipient R, that is, R' ← R + 2; or perhaps A doubles the number of segments of R, that is, R' ← 2 × R; or each stripe on A is a multiplier, that is, R' ← stripe(A) × R. The space of potential causal hypotheses is unbounded. One can use a generative model to express this infinite space using a small set of building blocks^37^. In this case, consider a probabilistic context-free grammar **G** with primitives stripe(A), spot(A), R, small integers 0, 1, 2, 3, and operations +, − and ×. Primitives stripe(A), spot(A) and R return corresponding numeric values. Operations such as + bind two numeric values and return a numeric value following the corresponding operation. Grammar **G** recursively samples these primitives to construct concepts (functions). Specifically, each operation primitive such as + can either bind numeric primitives or invoke another combination of operations, forming nested functions such as stripe(A) × (R − 1). Grammar **G** thus covers an infinite space of potential concepts and can be used to assign a probability distribution over this space (Methods). For a concept *z*, its prior probability is given by *P*_**G**_(*z*). As learners gather data D, they can check how likely it is that concept *z* will produce data D, known as likelihood *P*(D|*z*). According to Bayes’ rule, learners are then informed by the posterior *P*(*z*|D) ∝ *P*(D|*z*) × *P*_**G**_(*z*). While direct computation of this posterior is infeasible because the normalization term involves infinity, many methods exist to approximate this calculation^14,37–39^.

We build on this Bayesian-symbolic concept-learning framework to model conceptual bootstrapping. Specifically we use adaptor grammars (AG)^36^ as our generative grammar to assign prior probabilities. An adaptor grammar, by design, learns probabilistic mappings among subparts of a structure, capturing the intuition that when some concepts go together frequently, it makes sense to expect that the entire ensemble will be common in the future. Such a mechanism of caching concept ensembles and reusing them as a whole relaxes the context-free assumption of the context-free grammar **G** introduced above, and captures the essence of bootstrap learning: the effective reuse of learned concepts without the need to rediscover them every time it is used. Liang et al.^35^ extend adaptor grammars with combinatory logic, offering an algorithm for learning programmes that benefits from learning subprogramme sharing and reuse. Here we adapt the algorithm in Liang et al.^35^ to examine this cache-and-use mechanism as a process-level model of conceptual bootstrapping under resource constraints. Specifically, rather than sampling from a fixed set of primitives, we introduce a latent concept library that can be updated dynamically. Concept library *L* contains primitive concepts, as well as cached concept ensembles, weighted by how useful an ensemble has been (see below). Learners generate concepts using contents in library *L*, and adaptor grammar **AG** defines the probability that library *L* will generate concept *z* (Methods). This joint probability *P*(*z*, *L*) provides a prior *P*_**AG**_(*z*|*L*). We can then combine likelihood *P*(D|*z*) with this prior, yielding the posterior *P*(*z*|D, *L*).

The goal of inference is thus to infer the latent library *L* that can best account for learning data D. Following previous work suggesting that human learners make inferences by sampling from an approximate posterior rather than tracking the entire posterior space of possibilities^12^, we use known methods for sampling from Pitman–Yor processes^40^ such that, conditional on library *L* at any given moment, learners can make appropriate inferences about the probabilities of different explanations for new or salient events. In particular, we use Gibbs sampling (Methods), a Markov chain Monte Carlo method, over the joint distribution of concepts and libraries. At each iteration of Gibbs sampling, we sample a concept from this distribution *z* ~ *P*_**AG**_(*z*|*L*), and combine them with the likelihood function to determine concepts favoured by data. We then sample up to three favoured concepts and add them, as well as their subparts, to library *L* (caching; Fig. 1b), producing library sample *L'*. Note that in the next iteration, when sampling from *P*_**AG**_(z|*L'*), those added contents are used as if they were primitives (reuse; Fig. 1b) and therefore the learner can compose sophisticated combinations with rather few steps of composition (Methods).

This idea of a dynamic concept library is especially powerful when we take resource constraints into account. Taking the six observations in Fig. 1c for example, the ground truth concept involves different causal powers (maths operations) per agent feature. Therefore, trying to determine a concept consistent with all six observations is a challenging problem. However, if one looks at the first three pairs that involve only stripes (box bordered by solid lines, Fig. 1c), the learner may discover that stripes can multiply segments, R' ← stripe(A) × R. With this idea in mind and now looking at all six pairs, the learner may now manage to construct a nested concept R' ← (stripe(A) × R) – spot(A) that explains all observations by reusing the earlier concept as a subconcept. If we swap the presentation order and first show the learner the last three pairs in Fig. 1c (dashed-bordered box), the space of potential concept might overwhelm the learner, and without having cached any useful subconcepts, the full observation set might be just as confusing. Under our bootstrap learning model, individual learners could develop a concept library L* that is the result of two sequential episodes of posterior searching and caching. Provided that the first search phase leads to the learner caching the crucial building block stripe(A) × R, the second search phase is liable to result in their discovering and caching the ground truth, making this concept directly available when learners attempt to make generalizations and explicit guesses.

## Results

**Fig. 2:**
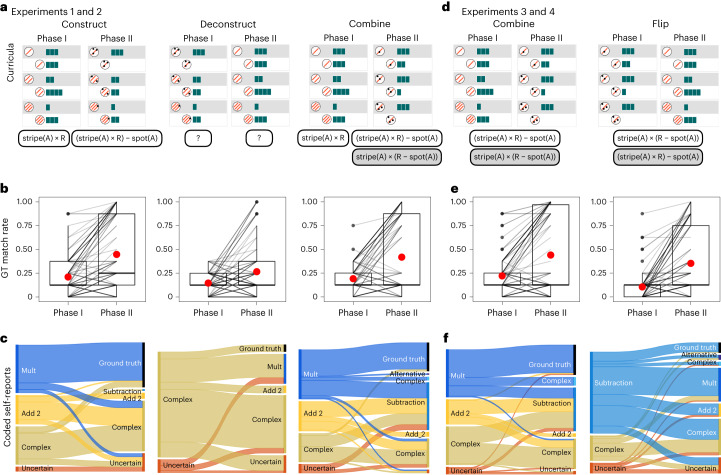
Experimental conditions and behavioural results. **a**, Curricula in Experiment 1. Experiment 2 is a feature counterbalance of this (Supplementary Information). Text boxes below each phase are data-compatible causal concepts; transparent text boxes are concepts favoured by the model, and shaded boxes for equally complex and data-consistent alternative concepts. **b**, Participantsʼ generalization accuracy (match to ground truth) in Experiments 1 and 2. Box plots show medians with major lines, first and third quantiles as bounds of box, smallest values within 1.5 times below the first quantile as minima, largest values within 1.5 times above the third quantile as maxima, and whiskers extending between box bounds and those values; red dots mark means. **c**, Coded self-reports in Experiments 1 and 2 (see Methods for coding scheme). For each curriculum, left bars for Phase I and right bars for Phase II. **d**, Curricula design in Experiment 3. Experiment 4 is a feature counterbalance of this and is available in Supplementary Information. **e**, Participants' match to ground truth in Experiments 3 and 4. **f**, Coded self-reports in Experiments 3 and 4.

Our bootstrap learning model predicts that a successful search for a complex target concept is heavily reliant on having good, previously learned abstractions. We test these model predictions using a two-phase causal learning and generalization task. In Phase I, learners observe three pairs of objects and their causal interactions (in fixed order, as illustrated in Fig. 2a), write down their guessed causal function and make generalization predictions on eight pairs of novel objects appearing in random order. Immediately after, in Phase II, learners observe three further pairs of objects and their causal interactions (with the previous three pairs still visible above), provide an updated guess to account for all six pairs and then make generalization predictions again on the same eight pairs as earlier, in a new randomized order (Methods).

### Curriculum-order effects in Experiments 1 and 2

Experiment 1 (*n* = 165) examined three curricula. Curriculum construct and deconstruct were as described in Fig. 1c and discussed above. We further included a combine curriculum that shares the same Phase I as in construct, but in Phase II keeps stripe(A) = 1 throughout (Fig. 2a), making it ambiguous about how stripe(A) × R and R − spot (A) should be combined. If people process Phase II with the cached subconcept from Phase I, we would expect to see R' ← stripe(A) × R – spot(A) more often than R' ← stripe(A) × (R –spot(A)). In follow-up Experiment 2 (*n* = 165) we flipped the roles of the stripes and spots of the agent object (Methods and Supplementary Information). While all key results replicate robustly in Experiment 2, we report per-curriculum collapsed results in analysis here for simplicity. First, we observed a significant difference in Phase II generalization accuracy—defined as ‘match to ground truth’—between the construct and deconstruct curricula. (Strictly speaking there are no wrong answers for the generalization tasks because they are all novel out-of-distribution pairs, such that any generalization prediction is justifiable under some inferred concept.) As illustrated in Fig. 2b, participants under the construct curriculum achieved an accuracy of 44.7 ± 38.3%, significantly higher than those with the deconstruct curriculum of only 22.6 ± 27.5% (*t*(1,717) = 8.13, *P* < 0.001, Cohen’s *d* = 0.4, 95% confidence interval (CI) [0.14, 0.24], chance accuracy 1/17 = 5.88%). The large standard deviations here imply a widespread individual difference in causal generalizations, demonstrating the openness and creativity of how people conceptualize causal relationships. Such individual difference crystallizes when looking at participants’ self-reports (Fig. 2c). For Phase II self-reported guesses, 37.8% of participants under the construct curriculum were classified as describing the ground truth (Fig. 2c) while under deconstruct condition only 6% did so (Wilcoxon test *z* = −5.75, *P* < 0.001, 95% CI [0, 0.0003], effect size = 0.5). A closer look at those self-reports revealed that, for those who induced that one feature multiplies in Phase I, 79% subsequently landed on ground truth in Phase II, showing a clear bootstrap learning trajectory. Recall that at the end of Phase II, in both construct and deconstruct curricula, participants had seen identical learning information (Fig. 2a) and hence this substantial difference in final learning performance coheres with our main claim that people reuse subconcepts to compose more complex ones. Merely observing evidence that favours a target concept is not sufficient to induce this concept.

The low matches with ground truth in self-reports in the deconstruct curriculum also reflect a strong garden-pathing effect^41^. We coded participants’ self-reports according to whether the content matches the ground truth, describes an operation such as multiplication, subtraction or addition and is uncertain or involves complex reasoning patterns drawing upon conditionals, positions of features or relative quantities (Methods). Notably, 89% of participants under the deconstruct condition came up with guesses classified as ‘complex’ in Phase I. For example, one participant wrote: “If there are more stripes than dots the stick is reduced in length. If there are equal stripes and dots then the stick stays the same. If there are more dots than stripes the stick increases in length.” This is a significantly higher proportion than the complex rule reported in construct Phase I (31.7%, Wilcoxon test *z* = −8.76, *P* <0.001, 95% CI [−1, −1], effect size = 0.8). The average length of Phase I guesses for the deconstruct curriculum was 168 ± 145 characters, also significantly longer than answers in the construct curriculum’s 112 ± 68.1 characters (*t*(168.09) = −3.76, *P* *<*0.001, Cohen’s *d* = 0.5, 95% CI [−85.65, −26.72]). These longer and more complex initial guesses appeared to influence the second phase of the experiment. In deconstruct Phase II, after seeing the simpler examples, 50% of complex-concept reporters either stuck with their initial complex guesses or embellished them even more, resulting in 48.7% complicated self-reported causal concepts in Phase II. Furthermore, only 24.8% of participants in Phase II of the deconstruct curriculum described that one feature multiplies, significantly lower than the 40.2% of construct curriculum participants after Phase I (Wilcoxon test *z* = −2.46, *P* = 0.01, 95% CI [0, 0.0001], effect size = 0.3). These results show that people frequently fall prey to learning traps in which initial complex examples prevent them from arriving at the ground truth^13,42^. Again, this pattern is consistent with the hypothesis that participants reuse their own Phase I ideas to bootstrap learning in Phase II.

Finally, participants under the combine condition overwhelmingly favoured ground truth over the alternative, despite these being equally complex and compatible with the data. In Phase II self-reports, 24.5% of participants under the combine condition reported the ground truth, with only one reporting the alternative concept (0.94%; Fig. 2c). Among these Phase II ground-truth reporters, 92.31% concluded that one feature multiplies in Phase I, aligning with our predictions that people reuse the Phase I learned concept as a primitive in Phase II. Interestingly, the Phase II generalization accuracy of the combine curriculum (41.7 ± 38.5%) did not differ significantly from that in the construct curriculum (44.7 ± 38.3%, *t*(1,702) = 1.25, *P* = 0.2). We further categorized a participant as responding according to the ground truth or the alternative concept if more than six out of the eight generalization predictions matched the corresponding concept. Here, 31 participants responded according to the ground truth (29%) and only one according to the alternative concept (0.01%, *χ*^2^(1) = 28.1, *P* < 0.001, Cramer’s *V* = 0.94), suggesting that the tendency of cache and reuse leads to systematic favouring of certain concepts over alternatives of the same level of accuracy and complexity.

### Biases in compositional form in Experiments 3 and 4

Results of the combine curriculum appear to support the idea that people reuse previous construction as conceptual primitives. However, it could also be compatible with the idea that people simply ‘glued’ the two subconcepts together additively—that is, (stripe(A) × R) + (− spot(A)) is logically equivalent to the ground truth. Furthermore, this ‘multiply-first’ function fits more naturally with the conventional order of mathematical operations in which multiplication is performed before addition in the absence of parentheses. To disentangle these concerns, we further designed a new curriculum, termed flip, which swaps Phase I and Phase II of combine (Fig. 2d). In this flip curriculum, if people reuse the concept they inferred in Phase I as a conceptual primitive in Phase II, they should conclude R' ← stripe(A) × (R – spot(A), the data-consistent alternative not favoured by the combine condition. If people instead use addition as their default or dominant compositional mode, then in flip Phase II we would expect that they will still favour the original ground truth. Experiment 3 (*n* = 120) tested this flip curriculum, together with the combine curriculum as in Experiment 1, using material exactly as shown in Fig. 2d. Experiment 4 (*n* = 120) reversed the causal powers between stripe and spot features but otherwise replicated Experiment 3 (Methods and Supplementary Information).

We found that people indeed favoured ground truth less often in the flip curriculum (Fig. 2e,f). Generalization accuracy, here defined as match to the original ground truth, for participants in flip Phase II was 35.2 ± 34.3%, while participants in combine achieved 44 ± 41.8% (*t*(1,881.9) = 3.93, *P* < 0.001, Cohen’s *d* = 0.2, 95% CI [0.04, 0.13]). In addition, only 8.7% of participants in the flip curriculum reported ground truth in Phase II, compared with 25.4% under the combine condition (Wilcoxon test *z* = −3.46, *P* < 0.001, 95% CI [0, 0.0001], effect size = 0.3). These results are in line with our previous finding that constructing, caching and later reusing the key subconcept is crucial for acquiring the complex target concept.

However, further examination suggests that the drop in synthesizing ground truth in flip was not primarily driven by turning to the alternative. Participants’ generalization accuracy in terms of matching the alternative concept was 28.8 ± 17.3%, lower than the level of agreement with the predictions of the original ground truth. As illustrated in Fig. 2f, five participants in flip Phase II reported the alternative concept (2.08%) in comparison with 16.7% guessing the ground truth (*χ*^2^(1) = 27.2, *P* < 0.001, Cramer’s *V* = 0.8). This suggests that additive compositional form is still quite a prevalent inductive bias, and it interacts with sequential bootstrap learning in phased reasoning tasks. Putting it another way, people may be choosing which phase to chunk according to their inductive bias on compositional form, and this might override the order in which evidence was actually presented in the experiments.

In our experimental interface, at the end of Phase II all six pairs of learning examples were available on the screen and participants could freely scroll up and down to revisit any earlier pairs. Such revisiting could induce orders of cache and reuse that are different from those designed by the experimenters. In fact, since we encouraged participants to synthesize causal relationships that can explain all six pairs, this may consequently encourage deliberate revisits. By revisiting evidence, in the flip curriculum a strong inductive bias on additive compositional form could lead to preferring ground truth over the alternative. In the deconstruct curricula in Experiments 1 and 2, some participants may have revisited Phase I after observing Phase II and thereby discovered the ground truth accordingly, reflected by the slight increase in Phase II generalization accuracy compared with Phase I in deconstruct (Fig. 2b).

### Model comparison

We now examine predictions and simulations from a range of computational models, comparing their ability to reproduce participants’ generalization patterns. First we considered a bootstrap learning model based on adaptor grammars AG as described in Formalization. Model AG first processes Phase I learning examples, acquiring an updated library, and then processes Phases I and II altogether with the updated library. Next, to account for the fact that participants were able to scroll up and down and reaccess Phase I after reasoning about Phase II, we considered a variant of AG, adaptor grammar with reprocessing (AGR). This model mixes predictions ${\hat{y}}_{\to }$ from Phase I to II, and predictions ${\hat{y}}_{\leftarrow }$ from Phase II to I, with a weight parameter *θ* ∈ [0, 1], acquiring a mixed prediction ${\hat{y}}_{r}\propto \theta \times {\hat{y}}_{\to }+(1-\theta )\times {\hat{y}}_{\leftarrow }$. Hyperparameters' values in models AG and AGR were the same as in Liang et al.^35^. From the estimated posterior libraries, we can collect a large number of generated concepts. Since concepts here are functions specifying R' for any agent–recipient object pairs, evaluation of these concepts on novel object pairs and marginalization on these predictions give a distribution of R' for novel object pairs (Methods).

For comparison, we examined a ‘rational rules’ (RR) model based on Goodman et al.^37^. This model assumes the same conceptual primitives as the adaptor grammar models but uses a probabilistic context-free grammar for prior concepts, as specified by grammar **G** in Formalization (see also Methods). Because we evaluate models using generalizations, we also implemented several subsymbolic models capable of generalization but not explicit rule guesses. Here we included a similarity-based categorization model (Similarity)^43^, a linear regression model (LinReg) and a multinomial regression model (Multinom). We further considered a Gaussian process regression (GpReg) model with radial basis function kernels (one per feature), because these models exhibit human-like performance in function learning and few-shot generalizations^44,45^. For the categorization and regression models, parameters were fitted to the learning examples predicting R' using stripe(A), spot(A) and R. We then made predictions about the novel objects with those fitted models, and evaluated model predictions in terms of their log-likelihood (LL) of producing participants’ predictions (Methods).

**Fig. 3:**
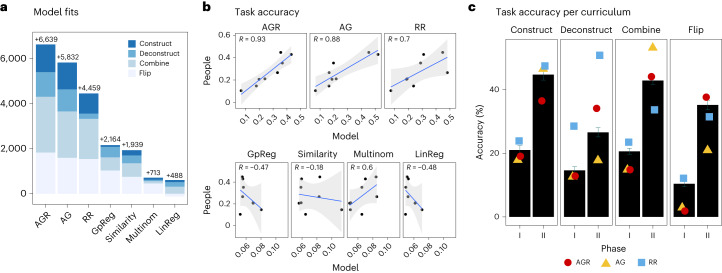
Modelling results. **a**, Model fit (total log-likelihood) improvement over random baseline (*y* = 0), log scale. **b**, Generalization accuracy according to curriculum and phase. *x* axis, model predictions; *y* axis, people’s predictions; error bands denote 95% CI. **c**, Generalization accuracy between participants' predictions (black bars, mean values ± SEM) and four symbolic models; *n*(construct) = 107, *n*(deconstruct) = 117, *n*(combine) = 220, *n*(flip) = 126.

Figure 3a shows each model’s improvement over a baseline model of random selection, Δ_model_ = LL_model_ − LL_random_. Model AGR achieves the greatest improvement, with the three Bayesian-symbolic models (AGR, AG and RR) easily outperforming similarity-based or regression models. With fitted model parameters, Fig. 3b plots generalization accuracy in each phase for each curriculum between model and people. In line with overall model fits, AGR best predicts people’s performance across all cases and the non-symbolic models fail to match people’s predictions.

Notably, while model RR can learn that some primitives are more common or useful than others, it is unable to discover and reuse concepts, as illustrated in Fig. 3a. We further plot generalization accuracies for models AGR, AG and RR against behavioural data in Fig. 3c, showing that model RR fails to reproduce the curriculum-order effects between construct and deconstruct curricula. This is because model RR is likely to have landed on the ground truth after seeing all the data, even for the deconstruct curriculum, and thus deviates from how people process phases of information. Model AG, on the other hand, is defeated by the learning trap because many people were exhibiting no accuracy improvement in Phase II relative to Phase I. Model AGR mixes model AG with some reprocessing and is therefore able to capture participants’ modest improvement in deconstruct Phase II generalizations. Furthermore, model RR achieves lower accuracy than people in the combine Phase II because it assigns as much posterior probability to the intended ground truth as to the equivalent-consistent alternatives.

**Fig. 4:**
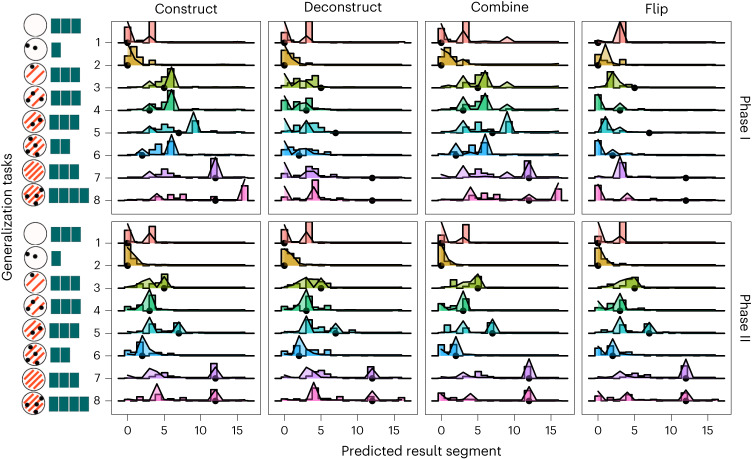
Generalization predictions by people (bars) and the best-fitting AGR model (area). Rows of panels are shown for experimental phases, columns for conditions. In each panel the *x* axis indicates predicted number of segments (0–16) and the *y* axis tasks ordered for analysis.

Figure 4 shows the best-fitting AGR model’s predictions in each generalization task, with participant data showing a close match. We note one interesting discrepancy in generalization task 1, which asked about an agent with no spots or stripes: while many participants predicted the disappearance of segments, because R' ← stripe(A) × R and 0 × 3 = 0, many participants also predicted that the resulting number of segments would remain the same. This could be due to participants concluding that absent features meant that nothing would happen. Future work could investigate how people reason about these kinds of edge cases.

Overall, the adaptor grammar models AG and AGR provided a much better account of people’s behavioural patterns in the experiments than the other models we considered. More generally, this means that both curriculum-order and garden-pathing effects exhibited by people can be explained as consequences of a cache-and-reuse mechanism expanding the reach of a bounded learning system. Critically, these phenomena cannot be explained by either a standard Bayesian-symbolic model out of the box or familiar subsymbolic categorization models, demonstrating that a cache-and-reuse mechanism is central to human-like inductive inference to compositional concepts.

## Discussion

We propose a formalization of bootstrap learning that supercharges Bayesian-symbolic concept-learning frameworks with an effective cache-and-reuse mechanism. This model replaces a fixed set of conceptual primitives with a dynamic concept library enabled by adaptor grammars, facilitating incremental discovery of complex concepts under helpful curricula despite finite computational resources. We show how compositional concepts evolve as cognitively bounded learners bootstrap from earlier conclusions over batches of data, and how this process gives rise to systematically different interpretations of the same evidence depending on the order in which it is processed. Being a Bayesian-symbolic model, our approach accounts for both the causal concepts people synthesized and the generalization predictions they made.

People often exhibit a general path dependence in their progression of ideas^46^. We show that this follows naturally when a bootstrap learner progresses in a space of compositional concepts, constructing complex ideas ‘piece by piece’ with limited cognitive resources. Crucially, we focus on how reuse of earlier concepts bootstraps the discovery of more complex compositional concepts using sampling-based inference. This builds on other sampling-based approximations to rational models^7^ that demonstrate how memory and computational constraints create focal hypotheses in the early stages of learning, and impair a learner’s ability to accommodate data they later encounter^13,38^. Going beyond this earlier work, we show how people exceed their immediate inferential limitations via reuse and composition of earlier discoveries through an evolving library of concepts. Our proposal also relates to the observation^47^ that amortized inference can explain how solving a subquery improves performance in solving complex nested queries. While our model instantiates reuse in a compositional space by caching conceptual building blocks in a latent concept library, there is potential to explore the connection between our formalization with amortized inference in terms of how reuse of partial computation might shape the approximation of the full posterior.

We also offer additional process-level explanations of why and how people often develop diverse understandings of the same evidence. People are known to develop biased interpretations of features^48^, and fall easily for various learning traps in category-based generalization related to selective attention or assumptions about stochasticity and similarity^42^. Jern et al.^49^ argued that different evaluations of the same evidence are due to different prior beliefs held by people. Tian et al.^33^ corroborated the premise that, equipped with different concept libraries, people can derive different solutions to the same problem set. Our formalization, however, demonstrates that markedly different conceptualization of the same evidence can arise among learners with the same learning mechanisms and even the same priors, systematically deviating from a normative approach to library learning. Note that our experiments tested causal learning and generalization in abstract settings rather than over subjective opinions such as political attitudes, and therefore serve as a friendly reminder that an objective interpretation is not guaranteed to prevail, even among capable cognizers scrutinizing the same data.

This interaction between our evolving concepts and our trajectory through the environment they seek to reflect lends itself to several interesting future directions. Culbertson and Schuler^50^ reviewed children’s performance in artificial language learning and stressed that learning is tightly bounded by cognitive constraints. We further found that inductive biases, such as those about the compositional forms we identified in Experiments 3 and 4, shape the order in which people process information. That is, rather than passive information receivers, it seems far more plausible that people have inductive biases of attention and action that shape how they select which subset of a complex situation to process first, and then build on that to make sense of the whole picture. Future work may extend our framework to active learning scenarios to study such information-seeking behaviours and self-directed curriculum design patterns in the domain of concept learning^51^. Moreover, cache and reuse is a useful way to refactor representations. Liang et al.^35^ introduced a subtree refactoring method for the discovery of shared substructures, providing natural future extensions for studying refactoring as a cognitive inference algorithm involved in the development of concepts^52^.

Recent research in neuroscience is starting to unravel how the brain may perform non-parametric Bayesian computations and latent causal inference^53^, and has uncovered representational similarities between artificial neural networks and brain activity^54,55^. Along these lines, neural evidence for the reuse of computational pathways across tasks^56^ would seem to support our thesis and further enrich our understanding of how the brain grows its conceptual systems and world models. One challenge for the symbolic framing adopted here comes from the fact that our conceptual representations are intimately tied in with their embodied sensorimotor features and consequences^57^. We look forward to more integrated models that capture how symbolic operations of composition and caching interface with such deeply embodied representations.

Our current work has several limitations that future work could address. For instance, we assumed a deterministic likelihood function but this does not efficiently handle vague concepts such as the stick decreases or increases. A grammar and likelihood able to capture concepts that constrain rather than uniquely predict generalizations could capture a larger range of people’s guesses and predictions. Because, for simplicity, we did not include conceptual primitives for conditionals, our model could not express all of the ‘divide-and-conquer’ self-reports people made when attempting to make sense of overwhelmingly complex information. This would be a straightforward extension, achievable by either starting with more basic primitives or assuming an if-else base concept. Piantadosi^58^ argued that base primitives in combinatory logic are sufficient to ground any Turing machine-computable mental representation and computation. We used natural language-like base terms simply for computational and expressive convenience, and all of the base primitives and learned concepts we assumed can be decomposed into solely combinatory logic bases. In addition, there exist many options other than combinatory logic to formalize our tasks. If we view variable objects A and R as hard-coded primitives, for example, a first-order logic formalization could have sufficed. We, however, preferred combinatory logic for its convenience and flexibility in routing variables, because this makes it easier to share and reuse any generated programme. One furher limitation of our current model is that it does not handle forgetting by default, a critical feature of human memory and learning^59–61^. To extend our formalization to model lifelong learning, it would be important to incorporate a mechanism through which concepts are forgotten, either through decay or being overwritten or outcompeted^62^.

In sum, we argue for the central role of bootstrap learning in human inductive inference and propose a process-level computational account of conceptual bootstrapping. Our work puts forward cache and reuse as a key cognitive inference algorithm and elucidates the importance of active information parsing for bounded reasoners grappling with a complex environment. Our findings stress the importance of curriculum design in teaching, and to facilitate communication of scientific theories. We hope this work will inspire not only social and cognitive sciences, but also the development of more data-efficient and human-like artificial learning algorithms.

## Methods

All experiments were performed with ethical approval from Edinburgh University Psychology Research Ethics Committee (ref. no. 3231819/1). Preregistration for each experiment is available at https://osf.io/9awhj/. All participants gave informed consent before undertaking the experiments.

### Experiment 1

#### Participants

A total of 165 participants (118 female, mean age (*M*_age_) = 31.8 ± 9.9) were recruited from Prolific Academic, according to a power analysis for three between-subject conditions seeking at least 0.95 power to detect a medium-size (≈ 0.35) fixed effect. Participants received a base payment of £1.25 and performance-based bonuses (highest payment, £1.93). The task lasted 9.69 ± 4.47 min. No participant was excluded from analysis.

#### Stimuli

Agent object A was visualized as a circle that moved in from the left of screen and collided with recipient R (Fig. 1a). A varied in regard to its number of stripes and randomly positioned spots; R took the form of a stick made up of a number of cube-shaped segments. During learning, all feature values were between 0 and 3. The rule we used to determine the recipient’s final number of segments was R' ← stripe(A) × R – spot(A). Learning materials were as shown in Fig. 2a. For generalization tasks an arbitrary segment number (0–16) could be selected, putting a nominal eyes-closed floor level of performance at 1/17 = 5.88%. Generalization trials were selected via a greedy entropy minimizing search to select a set that well distinguishes between a set of hypotheses favoured by model AG (Supplementary Information). Live demonstrations are available at https://bramleylab.ppls.ed.ac.uk/experiments/bootstrapping/p/welcome.html, and preregistration at https://osf.io/ud7jc.

#### Procedure

Each participant was randomly assigned to one of the three learning conditions—construct, deconstruct or combine. After reading instructions and passing a comprehension quiz, participants went through experiment Phase I followed by Phase II. In each phase, a participant tested three learning examples in the corresponding phase as shown in Fig. 2a, each appearing sequentially and as ordered in Fig. 2a. Participants watched the animated causal interactions by clicking a ‘test’ button. Once tested, a visual summary of the learning example, including the initial and final state of the recipient, was added to the screen and remained visible until the end of the experiment. Following the learning stage, participants were asked to write down their guesses about the underlying causal relationships and to make generalization predictions for eight pairs of novel objects. Generalization trials appeared sequentially. Once a prediction had been made, that trial was replaced by the next. The pairs of generalization objects in Phases I and II were the same, but their presentation orders were randomized for each participant and in each phase.

### Experiments 2–4

Experiment 2 is a feature-counterbalanced replication of Experiment 1 using true rule R' ← spot(A) × R – stripe(A). A further 165 participants (118 female, *M*_age_ = 33.8 ± 10.1) who did not participate in Experiment 1 were recruited from Prolific Academic. The task lasted 9.8 ± 5.2 min. No participant was excluded from analysis. Payment scale (highest payment £1.95) and procedure were identical to those in Experiment 1. Stimuli and preregistration are available at https://osf.io/k5dc3 and in Supplementary Information. We conducted two-way analysis of variance to analyse the effect of feature counterbalancing and curriculum design on Phase II generalization accuracy. While both factors had significant main effects (curriculum design, *F*(2, 2) = 9.2, *P* < 0.001; feature counterbalancing, *F*(1, 2) = 8.5, *P* < 0.001), there was no significant interaction (*F*(2, 324) = 0.15, *P* = 0.9). This indicates that people may be treating stripe and spot features differently, but this difference does not markedly interfere with our results for curriculum design.

Experiment 3 recruited a further 120 participants (72 female, *M*_age_ = 35.4 ± 10.9) to test the combine and flip curricula in Fig. 2d. We initially recruited 165 ÷ 3 × 2 = 110 participants to match group size in Experiments 1 and 2, but were faced with an imbalance between the two curricula (combine, 47; flip, 63) due to the random number generator used by the experiment to assign participants. To even out the samples we recruited a further ten participants on Prolific Academic on the same day, all to the combine curriculum, and ensured that this extra batch did not include participants from Experiments 1 and 2 and the current Experiment 3. All 120 participants were paid at the same scale as in Experiments 1 and 2 (highest payment £1.85). The task lasted 10.7 ± 4.5 min. The procedure was otherwise identical to Experiments 1 and 2. No participant was excluded from analysis. Preregistration for this experiment is available at https://osf.io/mfxa6, and full stimuli available in Supplementary Information.

Experiment 4 was a feature-counterbalanced replication of Experiment 3. We recruited a further 120 participants (76 female, *M*_age_ = 34.0 ± 12.6) from Prolific Academic and who had not participated in Experiments 1–3. Here the roles of the stripe and spot features was reversed as in Fig. 2d. Participants were paid at the same scale as in Experiments 1–3 (highest payment £1.83). The task lasted 9.2 ± 4.4 min. The procedure was identical to that in Experiments 1–3. No participant was excluded from analysis. Preregistration is available at https://osf.io/swde5. As above, two-way analysis of variance on feature counterbalancing and curriculum design predicting Phase II generalization accuracy revealed main effects on both factors (feature counterbalancing, *F*(1, 1) = 15.12, *P* < 0.001; curriculum design, *F*(1, 1) = 11.1, *P* = 0.001), but no interaction (*F*(1, 236) = 0.77, *P* = 0.4). While people indeed treat stripe and spot features differently, our results for curriculum design hold for both experiments.

### Coding scheme

Two coders categorized participant self-reports independently. The first coder categorized all free responses, and 15% of categorized self-reports were then compared against those of the second coder. Agreement level was 97.6%.

We identified eight codes. (1) Ground truth: equivalent to the ground truth causal relation in each experiment; for example, “length is multiplied by the number of lines and then the number of dots is subtracted” (Participant 43, Experiment 1). (2) Alternative: equivalent to the alternative causal relation in each experiment; for example, “the dots are subtracted from the segments by their number and the number of lines is multiplied by the number of segments” (Participant 461, Experiment 3). (3) Comp: unclear or implicit about how two subcausal concepts should be combined; for example, “the lines multiply the segments and dots subtract them” (Participant 451, Experiment 3). (4) Add 2: add two segments to the recipient object under the assumption that nothing happens if the agent object’s feature value is 1 (stripe in Experiments 1 and 3, and spots in Experiments 2 and 4); for example, “adds two segments to the stick only if there are two or more stripes on the egg” (Participant 35, Experiment 1). (5) Mult: one feature of the agent object multiplies the recipient object; for example, “the number of stripes multiplies the number of segments” (Participant 59, Experiment 1). (6) Subtraction: one feature of the agent object is a subtractor to the recipient object; for example, “each spot on the egg removes one stick” (Participant 100, Experiment 1). (7) Complex: describe the stimuli without generalizing a rule, or report a different rule for each observation; for example, “three dots means that the sticks disappear, two dots means two sticks and one dot means add another stick” (Participant 161, Experiment 1); “if there are more lines than dots it will increase in size but if there are more dots than lines it will decrease in size; an equal number of dots and lines will results in no change” (Participant 134, Experiment 1). (8) Uncertain: not knowing, unsure or confused about the learning stimuli; for example, “I don’t have a clue!” (Participant 57, Experiment 1).

### Analysis

To visualize and analyse data we used R v.4.1.1 (for parametric statistical analysis) and the following packages: rstatix v.0.7.2 (for non-parametric statistical analysis and default settings), tidyverse v.1.3.1, ggplot2 v.3.3.5, ggpubr v.0.4.0 and ggridges v.0.5.3. The Sankey flow charts shown in Fig. 2 were generated using Python v.3.9.1 and package pySankey v.0.0.1, installed from https://github.com/anazalea/pySankey.

### Adaptor grammar models

#### Algorithm 1

AG(*τ*, *X*)

**Require:** Type *τ* = *t*_0_ → … → *t*_*k*_

**Require:** variables *X* = {*x*_0_, …, *x*_*n*_}

 Sample *λ* ~ *U*(0, 1)

** if** *λ* ≤ *λ*_1_ **then**        ⊳Construct new hypothesis

   z_L_ ~ {z|*t*(*z*)_output_ = *t*_*k*_}  ⊳Sample a term, for example, mult

*   r* ~ **r**^|*X*|^          ⊳Sample a router, for example, **SC**

*   i* ← |*t*(*z*_L_)|           ⊳Grow RHS branches

**   while** *i* > 0, **do**

*    X*' = *r*(*X*)          ⊳Get routed variables

    ${\tau}^{{\prime} }=t({X}^{{\prime} })\to t{({{\it{z}_{{\mathrm{L}}}}})}_{i-1}$   ⊳Get type constraints

    AG (*r*', *X*')          ⊳Compose recursively

*    i* ← *i* − 1

**   end** **while**

** else**              ⊳Fetch existing hypothesis

   Return ^*^z ∈ *C*_*τ*_ with probability *λ*_2_

** end** **if**

#### Causal programmes

Because adaptor grammar **AG** expects modular reuse of programme fragments, we formalize programmes in combinatory logic^63^. This solves the variable binding problem in the generation of functional programmes^64^ and is supported by recent work by Piantadosi^58^ arguing that combinatory logic provides a unified low-level coding system for human mental representations. We start with defining a basic set of terms and types relevant to the task. This choice is for explanatory convenience and does not undermine our method’s ability to grow new types and new basic terms. In combinatory logic, each term *z* is treated as a function and constrained by its input domain type and output codomain type, written in the form *t*_input_ → *t*_output_, with right association by convention. Here we default the last type *t*_*n*_ in a type *t*_1_ → … → *t*_*n*_ to be the output type. Letting agent and recipient objects be variables with type obj, we consider basic terms getSpot, getStripe and getSegment, each with type obj → int, term setSegment, with type obj → int → obj, and terms add, sub and mult, each with type int → int → int. The term getSpot_obj→int_ takes an object as input and returns the integer number of spots on this object. The term add_int→int→int_ takes two integers as input and returns their sum as output; and likewise for the other terms above. We additionally consider four primitive integers 0, 1, 2 and 3, because these are the quantities appearing in the learning examples. Conveniently, we use *t*(*z*) to read the type of term *z*. For example, *t*(getSpot) returns obj → int. In addition, combinatory logic utilizes router terms such as **B**, **C**, **S** and **I** for variable binding. For a tree-like structure [router, *z*_L_, *z*_R_], router **B** sends variable *x* first to the right-hand side *z*_R_ (RHS), and the result of this is then sent to the left-hand side *z*_L_(LHS). In other words, [**B**, *z*_L_, *z*_R_](*x*) is executed as *z*_L_(*z*_R_(*x*)). Similarly, router **C** sends *x* to the left then right, router **S** sends *x* to both sides, and router **I** is an identity function that returns an input as it is. For *n* input variables we concatenate *n* routers in corresponding order.

#### Programme generation

We employ a tail recursion for composing terms, as in Dechter et al.^29^, to efficiently satisfy type constraints. As demonstrated in Algorithm 1, for a given target type *τ* = *t*_o_ → …*t*_*k*_, and a set of input variables *X* = {*x*_0_, …, *x*_*n*_}, with probability *λ*_1_ (see equation (1)) it enters the construction step, and with probability *λ*_2_ (see equation (1)) it returns a term with type *τ* and adds this returned term to the cache (hence the Return^*^ in Algorithm 1). The construction step starts by sampling a left-hand-side term, LHS, whose output type is the same as the output type of *τ*, *t*_output_(*τ*), which is *t*_*k*_ because we default the last element in a type to be the return type.

Following the notation in Liang et al.^35^, let *N* be the number of distinct elements in a collection of programmes C, and *M*_z_ the number of times programme z occurs in collection C:

1 $${\lambda }_{1}=\frac{{\alpha }_{0}+Nd}{{\alpha }_{0}+| {\mathrm{C}}| },\quad {\lambda }_{2}=\frac{{M}_{{\mathrm{z}}}-d}{| {\mathrm{C}}| -Nd}.$$

Hyperparameters *α*_0_ > 0 and 0 < *d* < 1 in equation (1) control the degree of sharing and reuse. Because *λ*_1_ is proportional to *α*_0_ + *Nd*, the smaller *α*_0_ and *d* are the less construction and more sharing we have. Similarly, because *λ*_2_ is proportional to *M*_z_, the more frequently a programme is cached the higher weight it acquires, regardless of its internal complexity. This definition of *λ*_2_ instantiates the idea of boostrapping—the prior generation complexity of a cached programme is overridden by its usefulness in regard to composing future concepts. At its core, AG reuses cached programmes as if they were conceptual primitives.

For simplicity, we assumed a flat prior initially such that terms sharing the same types have the same prior probability. Based on how many variables are fed to this stage, |*X*|, it then samples a router **r** of corresponding length from the set of all possible routers **r**^|*X*|^. This again is assumed to be a uniform distribution. For example, two variables correspond to 4^2^ = 16 routers {**BB**, **BC**, **BS**, **BI**, …}, and the probability of sampling each router is 1/16 = 0.0625. Router **r** then sends input variables to the branches. Now, the target type for the right-hand side of the tree is fully specified because it has all the input types (routed by **r**) and a required output type (to feed into LHS). Therefore, we apply the same procedure iteratively to acquire the right-hand-side subprogram RHS, returning the final programme [**r**, LHS, RHS]. The constructed programme [**r**, LHS, RHS] is then added to the programme library $L$ (caching). Note that, after caching, the counter for a term *z* in library *L* could change. That is, *M*_z_ in equation (1) is updated and preference for useful terms will then play a role in future programme generation.

#### Inference

Given this probabilistic model, we face the challenge of efficiently approximating a posterior distribution over latent programmes. Here we use known methods for sampling from Pitman–Yor processes^35,40^ such that, conditional on a programme library at any given moment, learners can make appropriate inferences about the probabilities of different explanations for new or salient events. This can be done via Gibbs sampling^65^: for the *i*th iteration, conditional on the library from previous iteration L_*i*−1_, sample an updated library L_*i*_ and add it to the collection of samples.

During each iteration of Gibbs sampling, when searching for programmes consistent with learning data we adopted a breadth-first beam search under resource constraints. Because the search space grows exponentially as depth increases, we hypothesize that people are more likely to search shallowly than deeply. Therefore we draw generation depth *d* ∝ e^−*bd*^, where *b* is a parameter controlling the steepness of this exponential decay. With generation depth *d*, we first enumerate a set of frames, ${{{\mathcal{F}}}}$ where rather than applying Algorithm 1 recursively, we use typed programme placeholders for LHS. We then sample a frame from ${{{\mathcal{F}}}}$ according to frame generation probabilities. The sampled frame is then ‘unfolded’, replacing each placeholder with a programme of the required type from the current library, yielding a set of fully articulated programmes *M*. If any programme(s) M^*^ ⊆ *M* produce learning data with likelihood 1, we stop the search and sample *n* = 3 programmes to enrich the library; otherwise, we sample another frame from ${{{\mathcal{F}}}}$ and repeat. If no programmes are perfectly consistent with the data after checking every frame from ${{{\mathcal{F}}}}$, we return with a “Nothing found” marker and move to the next iteration. Because of memory constraints we were able to enumerate frames up to depth *d* = 2, but this can easily produce deeply nested concepts as a result of iterated caching and reuse. We ran a grid search over integers 0–10 for parameter *b* in e^−*bd*^ on top of other model-fitting procedures. When *b* = 0, depth *d* = 1 and 2 searches are equally likely, and as *b* increases the model prefers depth *d* = 1. The best-fitting *b* = 6, implying a stronger preference for depth *d* = 1 (see Supplementary Information for additional analysis on search depth).

Thanks to the comprehensive search–check–sample procedure, we expect our Gibbs sampler to approximate the true posterior quickly and without the need for extensive burn-in. Because extensive Gibbs sampling is computationally expensive, and there is little value to running more than a handful of steps, we further assume that learners perform very little search within each phase. We thus approximate the population-level library distribution by running 1,000 simulations for chains of length *h*. During model fitting we compared simulations for length *h* = 1, 2, 3, 4 and 5, and found that the best-fitting model runs on an *h* = 2 chain (together with depth weight *b* = 6), suggesting strongly bounded use of resources (see Supplementary Information for additional analysis on chain length).

#### Generalizations

We run the generative procedure of grammar **AG** using the sampled libraries to approximate distribution Dist_*M*_ over latent causal programmes, and make generalization predictions about new, partially observed data D^*^ = 〈A^*^, R^*^, ?〉, producing a predicted distribution Dist_P_ over generalizations. Because we compare our models with the aggregated behavioural data, we ran the generation process 10,000 times for a posterior predictive of generalization predictions that is reasonably representative of the population. Note that these implementations are needed to set up a fair comparison between models and aggregated participant data. While generation of 10,000 hypotheses is certainly computationally demanding, this is not required for a single participant and is only to enable us to approximate a population-level distribution.

### Rational rules model

Following previous work^37,66,67^, we implemented a probabilistic context-free grammar ${\mathbf{G}} =\{ {\mathrm{S}}, T,M,N, {\Theta} \}$, where S is the starting symbol, *T* a set of production rules, *M* a set of non-terminal symbols {*A, B, C, D*}, *N* the set of terminal nodes, and Θ the production probabilities. To retain a close match with the adaptor grammar’s initial concept library, we considered production rules as follows:

$$\begin{array}{l}\mathrm{S}\to \,{{\rm{add}}}\,(\mathrm{A},\mathrm{A})\,| \,\,{{\rm{sub}}}\,(\mathrm{A},\mathrm{A})\,| \,\,{{\rm{mult}}}\,(\mathrm{A},\mathrm{A})\\ \mathrm{A}\to \mathrm{S}\,| \,\mathrm{B}\\ \mathrm{B}\to \mathrm{C}\,| \,\mathrm{D}\\ \mathrm{C}\to \,{{\rm{stripe}}}\,\,| \,\,{{\rm{spot}}}\,\,| \,\,{{\rm{segment}}}\,\\ \mathrm{D}\to 0\,| \,1\,| \,2\,| \,3\end{array}$$

The pipe symbol | represents ‘or’, meaning that the symbol on the left-hand side of arrow symbol → can transform to either of the symbols on the right-hand side of →. As with the adaptor grammar models, we assigned uniform prior production probabilities: let Γ_I_ be the set of production rules all starting with I— that is, any production rule *γ* ∈ Γ_I_ is of the form I → K, where K can be any symbol in grammar $\bf G$, the production probability for each *γ* ∈ Γ_I_ is $\frac{1}{\vert {\Gamma }_{\mathrm{I}}\vert }$. Because grammar $\bf G$ can produce infinitely complex causal concepts, we fixed a generation depth of *d* = 40 in our implementation to cover the ground-truth concepts. If *d* is set too small, as for the same constraint we set in the AG models, $\bf G$ cannot land on the ground truth by design and therefore is less useful in model comparison^68^. As in the adaptor grammar models, we used a deterministic likelihood function to evaluate each concept generated by grammar $\bf G$, essentially discarding all generated concepts that fail to explain all the evidence. We set *n* = 100,000 to acquire good coverage of rules up to and beyond the degree of complexity seen in human responses. Generalization predictions are made following the same procedure as the adaptor grammar models: apply the approximated posterior rules with the partially observed data D^*^ = 〈A^*^, R^*^, ?〉 in generalization tasks, and marginalize over the predicted R'* as an approximated posterior predictive.

### Similarity-based model

Let *d*_l_ be a learning example data point, consisting of an agent, a recipient object and a result object, and *d*_g_ a generalization task data point, consisting of only an agent and a recipient object. Let stripe(*x*) be the number of stripes of object x, and we can measure the similarity between learning example *d*_l_ and generalization task *d*_g_ in terms of stripes by taking the absolute difference $| | {\mathtt{stripes}}{({\mathrm{A}})}_{{d}_{{\mathrm{l}}}}-{\mathtt{stripes}}{({\mathrm{A}})}_{{d}_{{\mathrm{g}}}}| |$, denoted by *δ*_stripes_(*d*_l_, *d*_g_). Taking all three features—stripes, spots and segments—into account, the feature difference Δ between learning example *d*_l_ and generalization task *d*_g_ can be measured by Δ(*d*_l_, *d*_g_) = *a* × *δ*_stripe_(*d*_l_, *d*_g_) + *b* × *δ*_spot_(*d*_l_, *d*_g_) + *c* × *δ*_segment_(*d*_l_, *d*_g_). With these measures we can define a similarity score

$${\sigma }_{{{{\rm{sim}}}}}({d}_{{\mathrm{l}}},{d}_{{\mathrm{g}}})={\mathrm{e}}^{-\Delta ({d}_{{\mathrm{l}}},{d}_{{\mathrm{g}}})}$$

such that the more similar *d*_l_ and *d*_g_ are found to be (smaller distance Δ), the higher the similarity ${\sigma }_{{{{\rm{sim}}}}}$. When the two data points share the same agent and recipient objects, similarity score ${\sigma }_{{{{\rm{sim}}}}}$ reaches its maximal value of 1. When making generalization predictions, this model first computes similarity score ${\sigma }_{{{{\rm{sim}}}}}$ between the current generalization task *g*_*i*_ with all the available learning examples {*l*_1_, …, *l*_*k*_}, resulting in ${\mathrm{S}}=\{{\sigma }_{{{{\rm{sim}}}}}({d}_{{{\mathrm{l}}}_{1}},{d}_{{{\mathrm{g}}}_{i}}),\ldots ,{\sigma }_{{{{\rm{sim}}}}}({d}_{{{\mathrm{l}}}_{k}},{d}_{{{\mathrm{g}}}_{i}})\}$. Now, for this generalization task *g*_i_, it mimics result (${d}_{{{\mathrm{l}}}_{k}}$) with confidence ${\sigma }_{{{{\rm{sim}}}}}({d}_{{{\mathrm{l}}}_{k}},{d}_{{{\mathrm{g}}}_{i}})$. Letting $n={\mathtt{result}}({d}_{{{\mathrm{l}}}_{k}})$, task *g*_*i*_ predicts $p(n)={\mathtt{result}}({d}_{{{\mathrm{l}}}_{k}})\times {\sigma }_{{{{\rm{sim}}}}}({d}_{{{\mathrm{l}}}_{k}},{d}_{{{\mathrm{g}}}_{i}})$. Marginalizing over all possible result segment values *n* gives the distribution over the result segment values predicted by task *g*_*i*_.

### Linear regression model

Let the number of stripes, spots and segments in each learning example be the independent variables, and the resulting stick length R' be the dependent variable. We fit a linear regression model after each phase of the experiment with formula

$${{\mathrm{R}^{{\prime}}} } \sim a\times {\mathtt{stripe}}({\mathrm{A}})+b\times {\mathtt{spot}}({\mathrm{A}})+c\times {\mathrm{R}}+\epsilon .$$

We made generalization predictions using fitted parameters and the requisite generalization task’s feature values. We rounded the predicted result segment number to the two nearest integers to match the required prediction output.

### Multinomial logistic regression model

We treated each potential result segment value as a categorical value (rather than continuous as in the linear regression case), and fit a multinomial logistic regression model to predict the probability of each result segment value using the same formula as that used in the linear regression model, with the nnet package (v.7.3) in R (v.4.1.1). By fitting the model we call the pred function to gather probabilistic predictions about the potential result segment values for each trial. We normalize this probabilistic prediction to ensure that this is a probabilistic distribution.

### Gaussian process model

Treating each learning example as three-dimensional input (stripes, spots and segments) with a one-dimensional output (result segments), we fit a Gaussian process regression model with radial basis function kernels, each per feature *x*_*f*_:

$$K\left({x}_{f},{x}_{f}^{{\prime} }\right)=\exp \left(-\frac{| | {x}_{f}-{x}_{f}^{{\prime} }| | }{2{\sigma }^{2}}\right).$$

We used the GPy package (v.1.10.0) in Python (v.3.9.1) to fit the model. Conditioning on the three-dimensional input for each generalization task, the fitted Gaussian process regression model outputs a Gaussian distribution over potential segment lengths ${{{\mathcal{N}}}}(\mu ,{\sigma }^{2})$. We then bin this distribution over the potential discrete segment values for comparison with empirical data.

### Cross-validation

We used cross-validation to evaluate models against behavioural data in generalization tasks on log-likelihood fits. To do this we collapsed data from all four experiments by curriculum *c*, retaining how many people (*n*) chose which segment number *y* ∈ [0, 16] in each task *i*, resulting in data ${{{\mathcal{D}}}}=\{{n}_{ciy}\}$. We then let each computational model generate a distribution *P*_c*i*_ over all possible segment numbers *Y* = {0, 1, …, 16} for task *i* in curriculum *c*. Because many model predictions are point estimates, or are centred on only a few segment numbers, we considered a trembling-hand noise parameter $h\in (0,\frac{1}{| Y| })$ such that, for probability distribution *P*(*Y*),

2 $${P}^{\,h}(Y=y)=\frac{P(Y=y)+h}{1+h| Y\,| }.$$

Essentially, we add noise *h* to each random variable in set *Y* to avoid 0 likelihoods. The denominator ensures that *P*^*h*^(*Y*) is still a probability. Different from softmax functions, *P*^*h*^(*Y*) stays close to the shape of *P*(*Y*) when *h* is small and therefore best maintains each model’s ‘raw’ degree of confidence on those one or two predictions. The log-likelihood of a model producing data $D$ is thus given by

3 $${\mathrm{LL}}=\mathop{\sum }\limits_{c={c}_{1}}^{{c}_{k}}\mathop{\sum }\limits_{i={t}_{1}}^{{t}_{j}}\mathop{\sum }\limits_{y={y}_{1}}^{{y}_{m}}\ln ({P}_{ci}^{\,h}(Y=y))\times {n}_{ciy}.$$

For each run of the cross-validation we hold out one curriculum *c*_test_, and fit the noise parameter *h* on the other three curricula using maximum-likelihood estimation with the optim function in R. Note that, for model AGR, an additional weight parameter *λ* is jointly fitted. We then compute LL_test_ on curriculum *c*_test_ with the fitted parameters. Summing over LL_test_ for all four curricula serves as the total log-likelihood fit LL for the model. As a baseline, choosing randomly yields ${\mathrm{L{L}}}_{{{{\rm{rand}}}}}=570\times 16\times \ln (\frac{1}{17})=-25,838.91$ because there were 570 participants, each completing 8 × 2 = 16 tasks and where in each task there were 17 potential responses (final stick lengths, including 0) to choose from. Any value smaller than LL_random_ is an improvement over an eyes-closed baseline.

### Reporting summary

Further information on research design is available in the Nature Portfolio Reporting Summary linked to this article.

### Supplementary information

Supplementary Information Supplementary Figs. 1–4, discussion and Tables 1–3.

Reporting Summary

## Data Availability

Data reported in this study are available on the Open Science Framework (https://osf.io/9awhj/).

## Code availability

Implementations of all the models above and analysis are freely accessible at https://github.com/bramleyccslab/causal_bootstrapping and https://osf.io/9awhj/.
