# Supplementary material for: A model of conceptual bootstrapping in human cognition
Source: Nat Hum Behav. 2023 Oct 16;8(1):125–36. doi: 10.1038/s41562-023-01719-1 (PMC11349578; doi:10.1038/s41562-023-01719-1)
Supplement: Supplementary file 1 — Supplementary Figs. 1–4, discussion and Tables 1–3. [file 41562_2023_1719_MOESM1_ESM.pdf]

nature human behaviour

Article

https://doi.org/10.1038/s41562-023-01719-1

A model of conceptual bootstrapping in human cognition

In the format provided by the authors and unedited

Contents

Supplementary experiment stimuli **3**

Feature counterbalancing stimuli 3

Generalization task selection 3

Secondary analysis **4**

Task completion time and self-evaluated difficulty 4

Self-reported certainty 4

Self-report length 5

Additional model comparison **5**

Cross validation 5

Individual fit 6

Prediction match 6

Search depth and chain length 6

Comparison with GPT-3 **7**

List of Figures

1 Stimuli used for feature counterbalancing experiments Exp. 2 and Exp. 4. 13

2 Top, participants' self-reported certainty. Bottom, input length in terms of number of characters in participants' self-reports. In both figures, N(Exp. 1) = N(Exp. 2) = 165, N(Exp. 3) = N(Exp. 4) = 120. Box plots show medians with major lines, first and third quantiles as bounds of box, smallest values within 1.5 times below the 1st quantile as minima, largest values within 1.5 times above the 3rd quantile as maxima, and whiskers extending between the box bounds and those values. 14

3 Log likelihood improvement over baseline for Gibbs chain length (1 to 5) and weight parameters (0 to 10). Best fitting parameters are chain = 2 and weight = 6. 15

List of Tables

1 GPT-3 guesses about causal relationships 10

2 Summary statistics of average task completion time in minutes (± standard derivation), and average self-reported task difficulty on a scale of 1 to 10 where 10 is the most difficult (± standard derivation). 11

3 Model comparison results 12

Supplementary experiment stimuli

Feature counterbalancing stimuli

In correspondence with the experiment design in Exps. 1 and 3, we used a set of feature counterbalanced stimuli in replication experiments Exps. 2 and 4 (Figure 1), swapping the causal power associated with stripes and spots. For a pair corresponding tasks between the initial (Exps. 1 and 3) and replication (Exps. 2 and 4) experiments, i.e., same curriculum, same phase, same ordering, if the agent object in the initial task has x stripes and y spots, the agent object in the replication task will show y stripes and x spots, and the number of recipient and result segments between the two tasks are identical.

Generalization task selection

The eight generalization tasks were initially selected for Exp. 1. We used an identical set for Exp. 3, and took their feature counterbalancing version for Exps. 2 and 4. For 5 possible stripe values (0-4), 5 possible spot values and 4 possible recipient segment values, there are $5 \times 5 \times 4 = 100$ possible Agent-Recipient pairs. As a starting point, we ran a version of model AG that allows up to two exceptions in phase I and four in phase II, resulting in a large group of candidate programs M. We then grouped this very large M into 216 equivalent classes. That is, for two programs $m_1, m_2 \in M$, if $m_1(a, r) = m_2(a, r)$ for all the 100 possible pairs, then m_1 and m_2 belong to the same equivalent class. We kept the shortest program m_s in each equivalent class to be the class label, and recorded the size of each equivalent class to be their weight. Next, after excluding the learning pairs, we ran a greedy maximization of expected information gain for the rest of the pairs. Precisely, we start with selecting the Agent-Recipient pair that best distinguishes all these 216 programs, and then incrementally select the next best, taking previously-chosen pairs into consideration. To measure how well a pair distinguishes between the programs, we compute the expected information gain (EIG) for this pair over all possible programs, taking the normalized program weights from

their corresponding equivalent classes as the prior:

$$\text{EIG}(m, d) = H(m) - H(m|d), \tag{1}$$

where $H(\cdot)$ is the Shannon entropy:

$$H(X) = -\sum_{x \in X} p(x) \text{log} p(x). \tag{2}$$

After running greedy maximisation over EIG, we settled on a list of ordered Agent-Recipient pairs. We then picked the top eight of them, and replaced a four-stripe, zero-dot agent wit the zero-stripe, zero-dot agent because we were curious about how people would react to extreme feature values. This led to the eight generalization trials shown in Figure 4 in the main text. The feature counterbalanced version of them is available in Figure 1.

Secondary analysis

In the experiments, during each learning phase, after participants wrote down their best guesses about the causal relationships, we asked them how uncertain they were about the guesses. After completing all the tasks, in the debrief stage, we further asked participants how hard they found the entire experiment to be.

Task completion time and self-evaluated difficulty

Table 2 summarizes average task completion time and participants self-evaluated task difficulty. Overall, there is a positive correlation between task completion time and how hard people found the task to be. People generally spent more time in the *de-construct* condition, and found this curriculum a lot harder than the other curricula.

Self-reported certainty

Figure 2 (top) summarizes participants self-reported certainty in each phase. Curricula-wise, participants in *combine* and *de-construct* curricula reported growing certainty from Phase I to Phase II, while those in *construct* and *flip* demonstrated a drop. Participants following the *de-construct* curriculum reported lower certainty

throughout the experiments, in line with their lowest accuracy performance and highest self-evaluated task difficulty. The growth of certainty in the *combine* curriculum is intriguing, because in Phase II participants has no decisive information on how the two sub-concepts should be combined, yet they were quite confident about the composite concept they concluded. A reverse trend is found in the *flip* curriculum, which swaps Phase I and II information as in *combine*, again showing a strong interaction between inductive biases and sequential cache-and-reuse.

Self-report length

We reported that participants in the *de-construct* Phase I provided significantly longer self-reports than other conditions. Here, Figure 2 (bottom) visualizes the average self-report length in each phase for each curriculum per experiment. For self-reports in the *de-construct* curriculum, there is a sharp drop in self-report lengths from Phase I to Phase II, indicating that people turn to more consolidated answers after seeing simpler answers. In Exp. 1, self-reports in *de-construct* Phase II is still longer than *construct* Phase II and *combine* Phase II, reaffirming that people may be stuck in learning traps of their initial complicated guesses. In Exp. 2, however, *de-construct* Phase II self-reports were shorter than those in the other two curricula, due to a large proportion of people reported only the subtraction rule in this phase.

Additional model comparison

Cross validation

Table 3 lists all model comparison results in detail. Following the specifications in the main text, columns Construct, De-construct, Combine and Flip contain cross validation results on each corresponding held-out curricula. The Total NLL (short for negative log likelihood) column sums over the four curricula. The Improvement column takes each model's total NLL, and subtracts the Random baseline model's total NLL. All numbers in the above mentioned columns are log likelihoods, and a change in unit 1 reflects exponential scale of difference.

Individual fit

Column "N best fit" in Table 3 is the number of participants best fitted by the corresponding model. To evaluate this, for each participant, we compute the Bayesian information criterion (BIC) for all the models, and select the model with the lowest BIC to be the model that best fits this participant. We then compute how many participants each model best fits, serving as the "N best fit" measure. Model AGR best fits the most number of participants ($N = 150$), with model AG on a close match ($N = 141$), followed by the rational rules model ($N = 93$) and Gaussian Process regression ($N = 69$).

Prediction match

Since we forced a single-prediction per generalization task in the experiments, we compared how often a model's forced single-prediction matches people's most selected single-prediction in each task. To do so, for each model's distribution over predicted number of segments, we take the one with highest probability to be its single choice. There were no ties for all tasks and all the models we considered. Next, in the aggregated selection in each task from people, we took the most selected one. There were no ties either. We then compute how many of each model's single choice match with people's most favored option, being the "N match" measure. In total there are $8 \text{ task} \times 2 \text{ phase} \times 4 \text{ curriculum} = 64$ unique tasks. As Table 3 shows, model AGR and model AG match the most number of these forced single-predictions, N match = 45, constituting 70% of all tasks.

Search depth and chain length

For search depth, we sample generation depth d from a distribution following exponential decay controlled by a base parameter b, $d \propto e^{-bd}$, following the assumption that people search as shallow as they can. We ran a grid search over integers 0-10 for parameter b in e^{-bd} on top of other model fitting procedures. For chain length, we compare simulations for length $h = 1, 2, 3, 4,$ and 5, each with 1,000 runs. As illustrated in Figure 3, shorter chains and stronger commitment to depth-1 search

together produce better fits overall. The best fitting parameter combination is depth weight $b = 6$ and chain length $h = 2$. Extremely short chain ($h = 1$), or shifting towards depth-2 search steps, produce less human-like model predictions.

Comparison with GPT-3

In response to a growing interest in large language models being able to solve reasoning tasks like people, we transcribed our experiment stimuli in natural language, and probed GPT-3 to provide its best guesses about the causal relationship between observed causal agent and recipient objects. Overall, while GPT-3 is able to produce responses that flows naturally, it lacks some crucial inductive biases such as the multiplicative operation, and hence cannot discover the ground truth rule as most people do. Furthermore, there is no evidence for bootstrap learning in GPT-3's responses to either of the four experiments. We presented a list of GPT-3's responses in Table 1. For each entry in Table 1, we provided a verbal description of the observed agent's effect on the recipient (see below), and asked the GPT-3 prompt to complete a guess about the underlying causal relationship. We listed the first guess we get from each test in Table 1. The verbal description for each causal interaction follows a template

```
Observation: a magic egg with one stripe, touching three segments
Resulting segments: three
```

where the corresponding number of stripes, spots, segments, and resulting segments faithfully reflect those used in the experiments. After inputting three observations in Phase I and six in Phase II, we provided a question

```
How are stripes and spots changing the segments?
```

The text-completion responses from GPT-3 after this input is then collected and presented in Table 1.

We further exploit this work flow to collect generalization predictions. To do so, after providing verbal description of observations, instead of asking the

above-mentioned question, we presented a generalization task like so:

```
Observation: a magic egg with no stripe and no spot, touching three
    red↪ segments
Resulting segments:
```

where the number of stripes, spots and segments are taken according to each generalization task used in the experiments. By doing so, GPT-3 auto-completes a number, serving as its generalization prediction in this generalization task. Using the free OpenAI API, we were able to collect not only a single prediction, but also the top four predictions with their corresponding log-probabilities. The sum of these top four predictions' probabilities $\mathbf{P}$ usually constitute above 98% of total probabilities. We then equally divide $1 - \mathbf{P}$ among the other possible segment numbers, leading to a posterior prediction over all the entire selection space. We computed log likelihood fits for this posterior on participant data, $NLL_{\text{GPT-3}} = -26642.5$, surprisingly, worse than the random baseline -25838.9.

Exp.	Cur.	Ph.	GPT-3 completion
1	construct	1	Stripes are adding one segment for each stripe, while spots are subtracting one segment for each spot.
1	construct	2	The stripes and spots are not changing the segments.
1	decon	1	The stripes and spots on the egg are not affecting the segments.
1	decon	2	The stripes and spots on the magic egg are changing the resulting segments by adding or subtracting from the number of segments that are touching the egg.
1	combine	1	The number of stripes on the egg determines how many segments the egg will touch. The number of spots on the egg does not appear to have any effect.

Exp.	Cur.	Ph.	GPT-3 completion
1	combine	2	The stripes and spots on the magic egg are changing the segments because they are touching different segments. The egg with one stripe and one spot is touching two segments, while the egg with one stripe and three spots is touching three segments.
2	construct	1	The stripes and spots on the magic egg are changing the number of segments that the egg is touching. The more stripes and spots there are, the fewer segments the egg is touching.
2	construct	2	The stripes and spots are changing the segments by making the egg more magical. The more stripes and spots there are, the more magical the egg becomes, and the more segments it can touch.
2	decon	1	The stripes and spots on the magic egg seem to be affecting the segments in a way that is not yet known.
2	decon	2	The stripes and spots on the magic egg are changing the segments by adding or subtracting from the total number of segments. If there is one stripe and one spot, it will add two segments. If there are two stripes and two spots, it will subtract four segments.
2	combine	1	The stripes and spots on the egg seem to be dividing the segments into smaller parts. The more stripes or spots there are, the smaller the segments become.
2	combine	2	The stripes and spots on the egg seem to be dividing the segments into halves.
3	combine	1	The stripes and spots on the magic egg are changing the segments by adding or subtracting from the total number of segments.
3	combine	2	The stripes are dividing the segments into thirds, while the spots are halving the segments.

Exp.	Cur.	Ph.	GPT-3 completion
3	flip	1	The stripes and spots are not changing the segments.
3	flip	2	The stripes and spots on the egg seem to be affecting the number of segments the egg is touching. If the egg has one stripe and one spot, it is touching two segments. If the egg has one stripe and two spots, it is touching one segment. If the egg has one stripe and three spots, it is not touching any segments.
4	combine	1	The stripes and spots on the magic egg are changing the segments by breaking them up into smaller pieces. The egg is essentially dividing the segments into smaller sections, which makes it easier for the egg to touch more segments at once.
4	combine	2	It appears that stripes and spots are both reducing the number of resulting segments.
4	flip	1	The stripes and spots are not changing the segments.
4	flip	2	The stripes and spots on the magic egg are changing the segments by adding or subtracting from the total number of segments. One stripe subtracts one segment, while one spot adds one segment.

Table 1

GPT-3 guesses about causal relationships

Experiment	Condition	Task time	Self-reported task difficulty
1	construct	8.8 (±4.2)	6.4 (±2.7)
	de-construct	11.2 (±5.1)	7.8 (±2.1)
	combine	9 (±3.6)	6.4 (±2.3)
2	construct	10.5 (±5.3)	6.8 (±2.6)
	de-construct	10.9 (±6.3)	8.5 (±1.9)
	combine	7.9 (±2.9)	7.1 (±2.5)
3	combine	10.5 (±4.5)	5.9 (±2.5)
	flip	10.8 (±4.6)	6.5 (±2.3)
4	combine	8.5 (±3.2)	5.9 (±2.3)
	flip	9.9 (±5.2)	6.9 (±2.4)

Table 2

Summary statistics of average task completion time in minutes (± standard derivation), and average self-reported task difficulty on a scale of 1 to 10 where 10 is the most difficult (± standard derivation).

Table 3

Model comparison results

Model	Const.	De-con.	Comb.	Flip	Total	Improv.	N best fit	N match
AGR	**-3617**	**-4212**	**-7487**	**-3884**	**-19201**	**6639**	154	**44/64**
AG	-3659	-4325	-7906	-4117	-20007	5832	**227**	42/64
RR	-3955	-5076	-8183	-4166	-21380	4459	45	37/64
GpReg	-4770	-4840	-9386	-4678	-23674	2165	56	11/64
Similarity	-4616	-4959	-9364	-4962	-23901	1938	9	13/64
Multinom	-4761	-5244	-9864	-5255	-25124	715	34	17/64
LinReg	-4758	-5106	-9652	-5834	-25350	489	24	12/64
Random	-4850	-5304	-9973	-5712	-25839	0	21	9/64

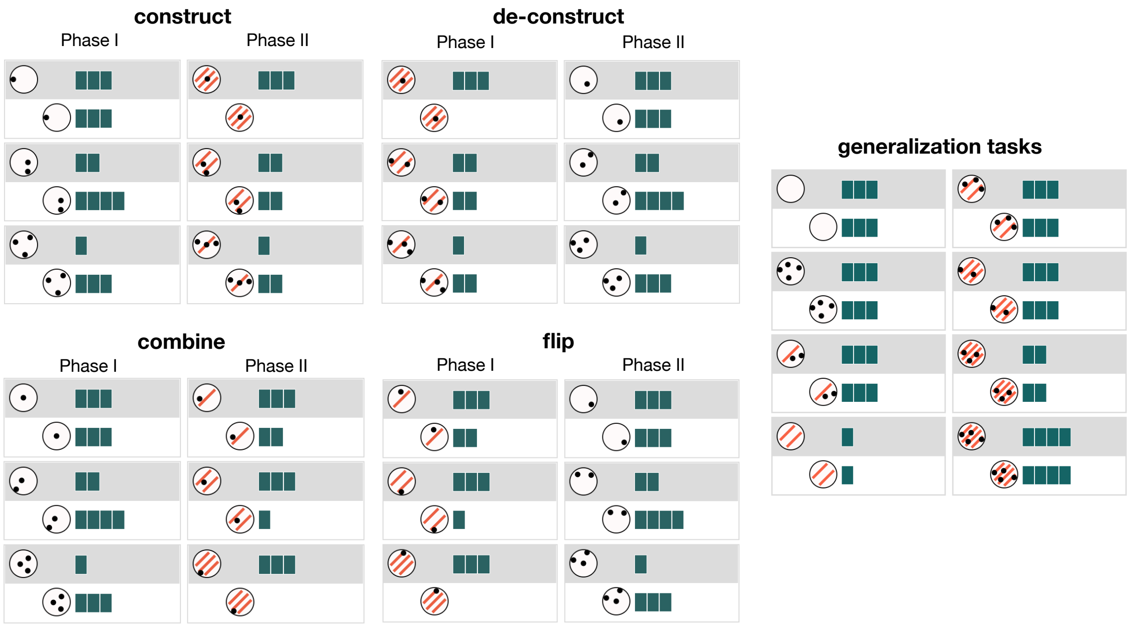

Figure 1. Stimuli used for feature counterbalancing experiments Exp. 2 and Exp. 4.

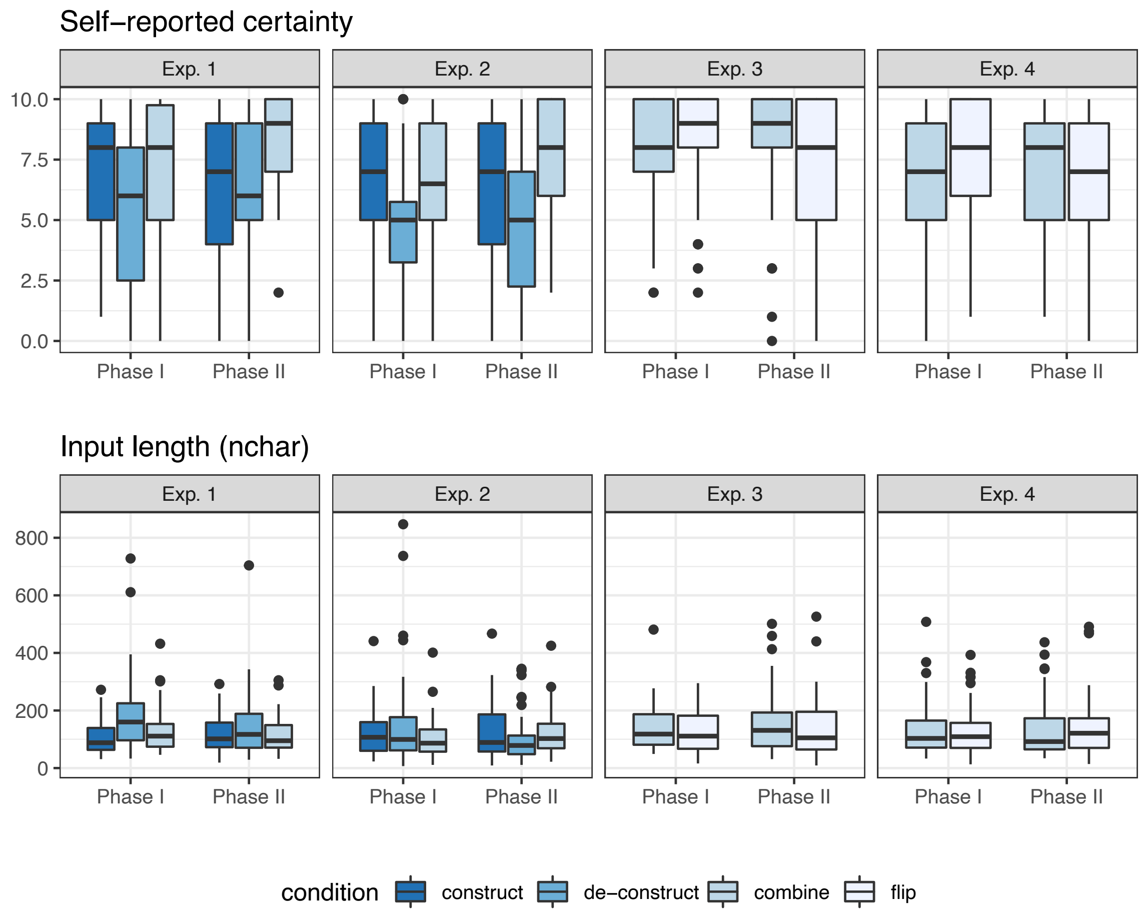

Figure 2. Top, participants' self-reported certainty. Bottom, input length in terms of number of characters in participants' self-reports. In both figures, N(Exp. 1) = N(Exp. 2) = 165, N(Exp. 3) = N(Exp. 4) = 120. Box plots show medians with major lines, first and third quantiles as bounds of box, smallest values within 1.5 times below the 1st quantile as minima, largest values within 1.5 times above the 3rd quantile as maxima, and whiskers extending between the box bounds and those values.

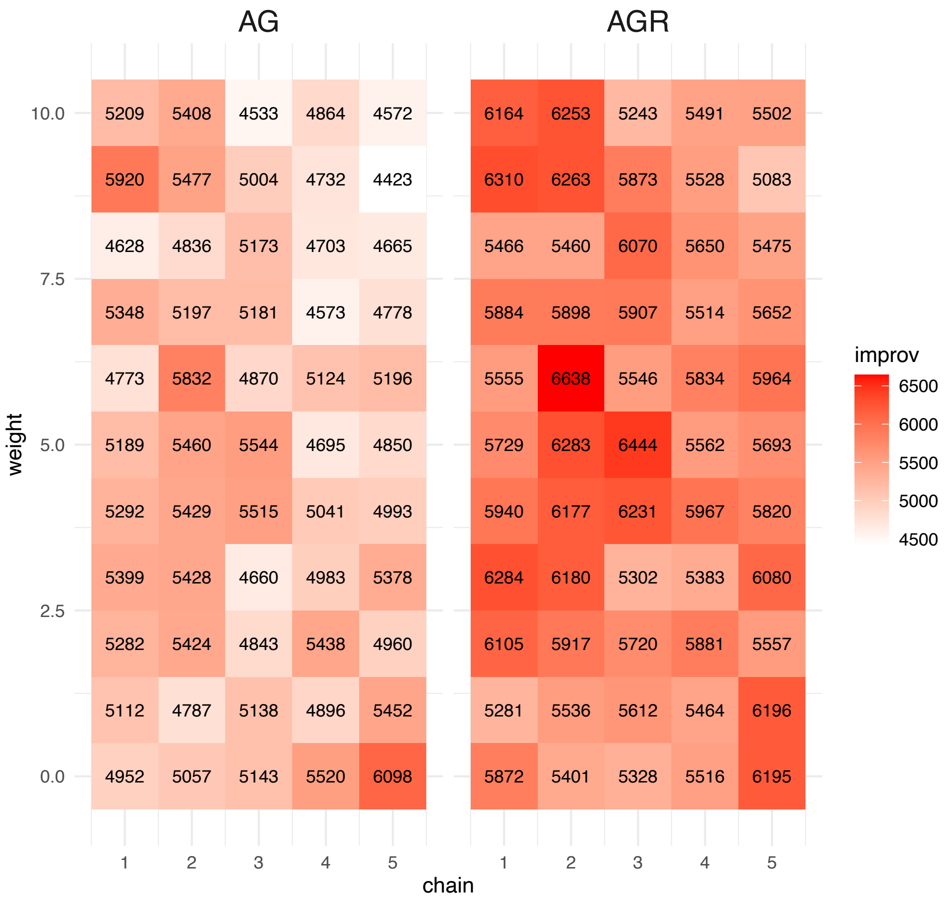

Figure 3. Log likelihood improvement over baseline for Gibbs chain length (1 to 5) and weight parameters (0 to 10). Best fitting parameters are chain = 2 and weight = 6.
