# Supplementary material for: A model of conceptual bootstrapping in human cognition
Source: Nat Hum Behav. 2023 Oct 16;8(1):125–36. doi: 10.1038/s41562-023-01719-1 (PMC11349578; doi:10.1038/s41562-023-01719-1)
Supplement: Supplementary file 2 — Reporting Summary [file 41562_2023_1719_MOESM2_ESM.pdf]

nature portfolio

Corresponding author(s): Bonan Zhao

Last updated by author(s): Aug 24, 2023

Reporting Summary

Nature Portfolio wishes to improve the reproducibility of the work that we publish. This form provides structure for consistency and transparency in reporting. For further information on Nature Portfolio policies, see our Editorial Policies and the Editorial Policy Checklist.

Statistics

For all statistical analyses, confirm that the following items are present in the figure legend, table legend, main text, or Methods section.

n/a	Confirmed	
☐	☒	The exact sample size (*n*) for each experimental group/condition, given as a discrete number and unit of measurement
☐	☒	A statement on whether measurements were taken from distinct samples or whether the same sample was measured repeatedly
☐	☒	The statistical test(s) used AND whether they are one- or two-sided<br>*Only common tests should be described solely by name; describe more complex techniques in the Methods section.*
☐	☒	A description of all covariates tested
☐	☒	A description of any assumptions or corrections, such as tests of normality and adjustment for multiple comparisons
☐	☒	A full description of the statistical parameters including central tendency (e.g. means) or other basic estimates (e.g. regression coefficient) AND variation (e.g. standard deviation) or associated estimates of uncertainty (e.g. confidence intervals)
☐	☒	For null hypothesis testing, the test statistic (e.g. *F*, *t*, *r*) with confidence intervals, effect sizes, degrees of freedom and *P* value noted<br>*Give P values as exact values whenever suitable.*
☒	☐	For Bayesian analysis, information on the choice of priors and Markov chain Monte Carlo settings
☒	☐	For hierarchical and complex designs, identification of the appropriate level for tests and full reporting of outcomes
☐	☒	Estimates of effect sizes (e.g. Cohen's *d*, Pearson's *r*), indicating how they were calculated

Our web collection on statistics for biologists contains articles on many of the points above.

Software and code

Policy information about availability of computer code

Data collection	Data was collected using an on-line experiment coded in JavaScript, HTML and CSS. Implementation for the experimental interface is available at https://github.com/bramleyccslab/causal_bootstrapping/tree/main/experiment under MIT license.
Data analysis	To visualize and analyze data, we used R version 4.1.1 and the following packages: nnet version 7.3, tidyverse version 1.3.1, ggplot2 version 3.3.5, ggpubr version 0.4.0, and ggridges version 0.5.3. The full analysis script is available at https://bramleylab.ppls.ed.ac.uk/experiments/bootstrapping/analysis.html. The Sankey flow chart is generated using Python 3.9.1 and package pySankey version 0.0.1 installed from https://github.com/anazalea/pySankey.<br><br>To implement our models and generate predictions, we used Python version 3.9.1. and the following packages: numpy version 1.20.2, pandas version 1.2.3, and GPy version 1.10.0. The full model implementation is available at https://github.com/bramleyccslab/causal_bootstrapping/tree/main/models under MIT license, and can be reached through https://osf.io/9awhj/.

For manuscripts utilizing custom algorithms or software that are central to the research but not yet described in published literature, software must be made available to editors and reviewers. We strongly encourage code deposition in a community repository (e.g. GitHub). See the Nature Portfolio guidelines for submitting code & software for further information.

Data

Policy information about availability of data

All manuscripts must include a data availability statement. This statement should provide the following information, where applicable:

- Accession codes, unique identifiers, or web links for publicly available datasets
- A description of any restrictions on data availability
- For clinical datasets or third party data, please ensure that the statement adheres to our policy

All data and analysis scripts are available on the Open Science Framework at the following link: https://osf.io/9awhj/

Human research participants

Policy information about studies involving human research participants and Sex and Gender in Research.

Reporting on sex and gender	Gender was collected based on self-report in the debrief stage of the experiment. No gender-related effects were detected.
Population characteristics	See above (Behavioural & social sciences study design).
Recruitment	Participants were recruited through Prolific Academic with criteria of being adult and English speaking. No other extra selection criterion.
Ethics oversight	All experiments were performed with ethical approval from Edinburgh University Psychology Research Ethics Committee (Ref No: 3231819/1). All participants gave informed consent before undertaking the experiments.

Note that full information on the approval of the study protocol must also be provided in the manuscript.

Field-specific reporting

Please select the one below that is the best fit for your research. If you are not sure, read the appropriate sections before making your selection.

☐ Life sciences ☒ Behavioural & social sciences ☐ Ecological, evolutionary & environmental sciences

For a reference copy of the document with all sections, see nature.com/documents/nr-reporting-summary-flat.pdf

Behavioural & social sciences study design

All studies must disclose on these points even when the disclosure is negative.

Study description	Double-blinded randomized control experiments where participants are randomly assigned to different stimuli, reported casual concepts they concluded from the stimuli, and then made generalization predictions and provided self-reports.
Research sample	We recruited 165 participants for Experiment 1 (118 Female, mean age ± SD 31.8 ± 9.9), according to a power analysis for three between-subject conditions seeking at least 0.95 power to detect a medium size (≈0.35) fixed effect. Another 165 for Experiment 2 (118 Female, mean age ± SD 31.8 ± 9.9) following the same size as Experiment 1, 120 for Experiment 3 (72 Female, mean age ± SD 35.4 ± 10.9), and 120 for Experiment 4 (76 Female, mean age ± SD 34.0 ± 12.6). Sample size for Experiment 3 was initially determined by the same per-condition sample size as in Experiments 1 and 2 (165 ÷ 3 × 2 = 110), but was faced with an imbalance between the two between-subject conditions due to the random number generator the experiment used to assign participants. To even out the samples, we recruited another 10 participants on Prolific on the same day for the one with fewer participants. Experiment 4 replicated the sample size as in Experiment 3.
Sampling strategy	Participants were randomly assigned to one of the several conditions in each experiment, according to a Math.random() javascript function upon landing at the experiment page.
Data collection	Data was collected on-line through desktop devices, and saved to an encrypted database on a server owned by the University of Edinburgh. No researcher was present when participants took the experiments. Experimental conditions are blinded both to the experimenter and the participants.
Timing	Experiment 1 was conducted on Dec 22, 2021, Experiment 2 on Nov 9, 2021, Experiment 3 on Dec 8, 2021, and Experiment 4 on Feb 16, 2022. Each experiment was launched at 10am Edinburgh local time and stopped when the planned number of participants had taken the study.
Data exclusions	No data was excluded from analysis.
Non-participation	No participants declined/dropped out.

Randomization	Participants were randomly assigned to each between-subject condition according to a random number generator in JavaScript.

Reporting for specific materials, systems and methods

We require information from authors about some types of materials, experimental systems and methods used in many studies. Here, indicate whether each material, system or method listed is relevant to your study. If you are not sure if a list item applies to your research, read the appropriate section before selecting a response.

Materials & experimental systems

n/a	Involved in the study
☒	☐ Antibodies
☒	☐ Eukaryotic cell lines
☒	☐ Palaeontology and archaeology
☒	☐ Animals and other organisms
☒	☐ Clinical data
☒	☐ Dual use research of concern

Methods

n/a	Involved in the study
☒	☐ ChIP-seq
☒	☐ Flow cytometry
☒	☐ MRI-based neuroimaging
